# Land application of industrial wastes: impacts on soil quality, biota, and human health

**DOI:** 10.1007/s11356-023-26893-7

**Published:** 2023-05-04

**Authors:** Shamali De Silva, Peter Carson, Demidu V. Indrapala, Barry Warwick, Suzie M. Reichman

**Affiliations:** 1 Environment Protection Authority Victoria, EPA Science, Macleod, VIC 3085 Australia; 2 School of Engineering, RMIT University, Melbourne, 3001 Australia; 3 Centre for Anthropogenic Pollution Impact and Management (CAPIM), University of Melbourne, Parkville, 3010 Australia; 4 School of Biosciences, University of Melbourne, Parkville, 3010 Australia

**Keywords:** Agriculture, Contamination, Emerging chemicals of concern, Groundwater, Review, Risk assessment, Soil amendment, Sustainable waste disposal

## Abstract

Globally, waste disposal options such as landfill, incineration, and discharge to water, are not preferred long-term solutions due to their social, environmental, political, and economic implications. However, there is potential for increasing the sustainability of industrial processes by considering land application of industrial wastes. Applying waste to land can have beneficial outcomes including reducing waste sent to landfill and providing alternative nutrient sources for agriculture and other primary production. However, there are also potential hazards, including environmental contamination. This article reviewed the literature on industrial waste applications to soils and assessed the associated hazards and benefits. The review investigated wastes in relation to soil characteristics, dynamics between soils and waste constituents, and possible impacts on plants, animals, and humans. The current body of literature demonstrates the potential for the application of industrial waste into agricultural soils. The main challenge for applying industrial wastes to land is the presence of contaminants in some wastes and managing these to enhance positive effects and reduce negative outcomes to within acceptable limits. Examination of the literature also revealed several gaps in the research and opportunities for further investigation: specifically, a lack of long-term experiments and mass balance assessments, variable waste composition, and negative public opinion.

## Introduction

Globally, waste disposal options such as landfill, incineration and discharge to water, are not preferred long-term solutions due to their social, environmental, political, and economic implications (Cameron et al. 1997). Expanding industrialisation, population growth, and growing consumer demand are placing stress on existing waste management strategies (Haynes 2009). Across the world, more sustainable ways of handling industrial waste through increased recycling and reuse alternatives are being considered. Thus, there is potential for increasing the sustainability of industrial processes by considering land application of wastes. Applying waste to land can have beneficial outcomes including reducing waste sent to landfill and providing alternative sources of nutrients for agricultural and other primary production systems (Pires and Martinho 2019; van Ewijk and Stegemann 2020).

Studies have shown that the functional benefits of waste application to land can range from increased plant growth and yield, to improved soil structure and alleviation of undesirable soil conditions such as acidity and erosion (Basu et al. 2009; Manoharan et al. 2010; Yunusa et al. 2006). Intensive agricultural practices are dependent on the routine use of fertilisers and liming agents to replenish nutrients and sustain high production. Utilising the beneficial properties of industrial wastes has the potential to provide alternatives to fertilisers and soil conditioners for farming systems. Using wastes instead of, or as a supplement to, commercial products could alleviate the financial pressures imposed on farmers by fluctuating global markets (ACCC 2008).

In addition to the potential benefits from applying wastes to agricultural landscapes, there are also significant potential hazards, including contamination of soils; groundwater; and human and animal food sources from contaminants present in the wastes. In many countries, the acceptance of industrial waste as a soil amendment in agriculture is highly contentious and limited by uncertainty about the effects of contaminants on soil, water, food, and, subsequently, human health (Dessalew et al. 2017; Kirchmann et al. 2017; Spark and Swift 2008). Consequently, the application of wastes as soil amendments must be in a way that ensures safe and sustainable outcomes.

In this review, we have summarised and assessed the scientific literature on the application of industrial wastes to arable land. The review addresses the waste applications of various industries, including foundries, smelters, coal-burning power plants, and agriculture. This review will not focus on biosolid application to land as it has been covered in several recent reviews e.g., Badzmierowski et al. 2021; Clarke and Smith 2011; Gianico et al. 2021; Jjemba 2002b; Lu et al. 2012; Ma and Rosen 2021; and Torri et al. 2017. For the purposes of this review, we considered ‘biosolids’ to be organic solids derived from human sewage treatment processes (EPA Victoria 2004); solid wastes that do not meet this criteria are referred to as ‘sludge’. The review aims to provide information and identify knowledge gaps in the literature on the practice of applying industrial waste to agricultural lands. Whilst there have been multiple reviews focusing on the application of particular industrial wastes to land, or for specific regions or chemical contaminants, there has not been a review synthesising knowledge globally across waste types. To our knowledge, this is the first wholistic review on the use of industrial wastes as soil amendments that synthesises information across waste types; soil characteristics; and environmental and human health risk. We have explored the dynamics between soils and waste constituents, as well as assessing the hazards and benefits associated with the application of wastes to land.

## Waste application to land

### Waste types

The wastes addressed in this review are broadly categorised as those produced by industrial facilities, such as mills, factories, agriculture, and power stations. In general, the literature addressing waste has predominantly focused on certain types of waste, in particular fly ash^1^ (Blissett and Rowson 2012; Ram and Masto 2014; Shaheen et al. 2014), rather than evenly covering the broad spectrum of wastes that could be potentially applied to land. Other literature covers wastewater (Johns 1995; Mittal 2006), a term which often refers to a combination of industrial water-based waste discharged into waterways from multiple industries or stored in lagoons. In most cases, the wastes investigated have been sourced directly from the producer and undergone tests in an untreated state, as such, in depth coverage of industrial treatments prior to application of wastes to soil is outside the scope of this review.

**Fig. 1:**
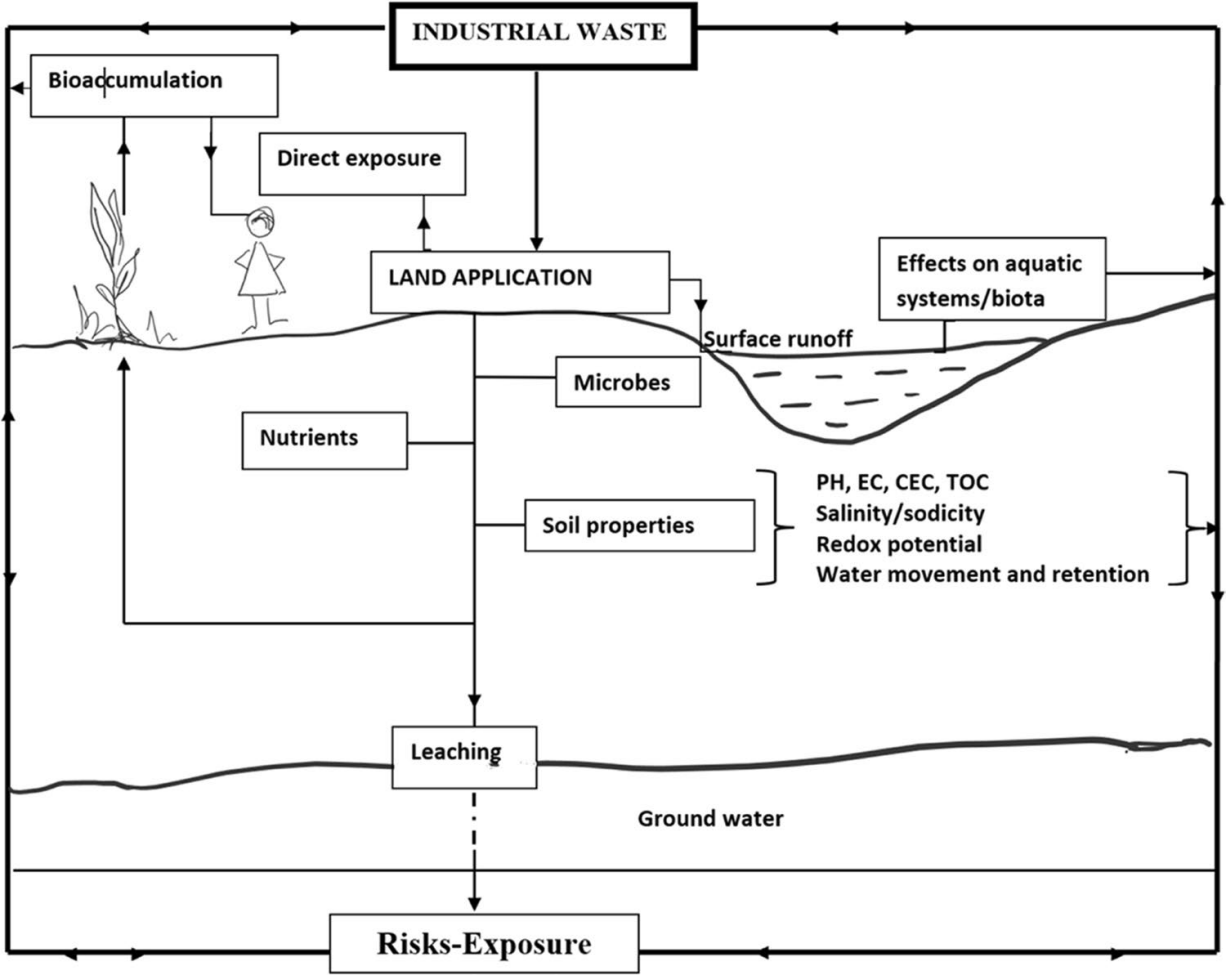
Conceptual diagram of the fate and behaviour of contaminants in industrial wastes applied to land

A summary of the wastes that have been identified as potential soil amendments, outlining the possible uses and risks of each waste type, is provided in Table 1. As listed in Table 1, wastes generated by industrial activities vary in their material composition and elemental constituents. Constituents, processing, chemical agents, batch number, and technology can have significant impacts on the resulting waste (Arvanitoyannis 2006; Luther 2011). Consequently, the development of procedures for waste application to land is complicated by the variations present in each batch and the type of waste. Furthermore, once wastes are applied to land, they interact with the soil often changing the physiochemical properties of the soil and waste, and thus, the potential impacts on the environment (Fig. 1).

**Table 1:** Selected industrial wastes and their soil amendment properties

Industrial waste	Waste category based on effects on soil	Waste effect on soil	Potential disadvantages	References
Cement and lime kiln	Soil pH	Increase pH High in Ca	Potential fugitive dust Highly caustic Variable composition May contain contaminants Low nutrient value (variable)	Allen et al. 2007
Coal combustion products e.g., fly ash (class C^1^ and F^2^)	Soil physical properties, soil pH, fertiliser	Modifies soil texture Water retention Increase pH Source of nutrients	Variable composition May be high in B May be high in salts May leach Se and As May contain contaminants (PCB and PAH^3^) Low C and N	Amiralian et al. 2012; Basu et al. 2009; Manoharan et al. 2010; Nalbantoğlu 2004; Shaheen et al. 2014; Yunusa et al. 2011; Yunusa et al. 2006
Foundry sand	Soil physical properties	Modifies soil texture Sorbent Water retention	May contain contaminants High Na Water retention	Dungan et al. 2006; Zhang et al. 2014
Pulp sludge	Soil physical properties, organic matter	Slope stabiliser Water retention, source of OM	Variable constituents Total C may not reflect availability Very low nutrient value (variable) May contain contaminants	Cameron et al. 1997; Tabassum et al. 2015; USEPA 2000
Red mud bauxite	Soil physical properties, fertiliser	CCE^3^ sorbent High Mn	May volatise ammonia	Brunori et al. 2005; Vlahos et al. 1989
Sugar beet lime	Soil pH, fertiliser	Increase pH High P potential sorbent	Increase pH High P	Blanco-Velázquez et al. 2019; Fares et al. 2014; Öğüt and Er 2015; Pacharane et al. 2021
Tannery waste	Fertiliser	Source of nutrients	High Cr (variable), Cu, Zn, Pb, Sr, and V High salt content	Alvarez-Bernal et al. 2006; Calheiros et al. 2008; Gowd et al. 2010; Kankaria et al. 2011 Nabavinia et al. 2015
Dairy effluents	Fertiliser, organic matter	Source of nutrients Source of organic matter	Highly variable quality Variable constituent May have high nutrient loadings May contain unsafe contaminants	Allinson 2008; Arvanitoyannis 2006; Yang et al., 1989 Habteselassie et al. 2006a; Habteselassie et al. 2006b; Hawke and Summers 2006; Sparling et al. 2001; Zaman et al. 2002
Potable water treatment residuals	Soil physical properties, fertiliser	Phosphorous binding Potential sorbent	Different materials have variable reactivity May contain As and radioactive isotopes	Zhao et al. 2018; 2010; Dayton and Basta 2001; Howells et al. 2018; Lombi et al. 2010; Mahdy et al. 2009, Turner et al. 2019; Nguyen et al. 2022
Wood ash	Soil physical properties, Soil pH, fertiliser, organic matter	Modifies soil texture Increase pH Source of nutrients Source of organic matter	May be high in B variable composition May have high nutrient loadings	Arvidsson and Lundkvist 2003; Demeyer et al. 2001; Moragues-Saitua et al. 2017; Nkana et al. 1998; Ohno and Erich 1990

1Class C fly ash originates from subbituminous and lignite coals. The composition consists mainly of calcium, alumina, and silica with a lower loss on ignition than class F fly ash (ASTM, 2005).

^2^Class F fly ash originates from anthracite and bituminous coals (ASTM, 2005).

^3^*PAH*, polycyclic aromatic hydrocarbon; *PCB*, polychlorinated biphenyls; *CCE*, calcium carbonate equivalent.

Soils are structurally diverse materials with a range of physiochemical properties. Soil attributes affected by waste amendments may include texture, bulk density, pH, water holding capacity, cation exchange capacity (CEC), exchangeable sodium percentage (ESP), organic matter (OM), and biological properties (Table 1) (Alvarez-Bernal et al. 2006; Mittra et al. 2005; Pandey and Singh 2010; Punshon et al. 2002; Yu et al. 2015). Soils amended with industrial wastes can improve soil physical properties (Dexter 2004; Eneje Roseta and Innocent 2012; Kaur and Sharma 2014; Killi et al. 2014; Tabassum et al. 2015). Soil conditions at waste application sites vary widely; therefore, soil is an important factor to incorporate when designing guidelines for applying wastes to land (Luther 2011).

### Waste pretreatment

Whilst applying industrial wastes to land can be considered a beneficial reuse, it is also a form of waste disposal. Waste application to land therefore, often requires authorisation by the relevant jurisdiction before the commencement of any industrial waste treatment and land application (e.g., State of Victoria 2021; British Standards Institute 2014).

The physiochemical properties of the untreated wastes can be a problem for its safe reuse (see Table 1). Thus, various treatment technologies for waste treatment can be used to minimise potential environmental impacts when wastes are applied to land (Table 2). Examples of treatment options include composting, rendering, anaerobic digestion, controlled incineration, nutrient extraction, and aerobic-activated sludge systems (Bandaw and Herago 2017). Composting is effective in breaking down waste and organic material and can kill some pathogens. Rendering involves mechanical, thermal, and chemical treatment of solid livestock waste e.g., slaughter waste and whole carcasses, to produce pelleted soil additives or animal feedstock such as meat and bone meal and tallow (fats and oils) (Mittal 2006). Anaerobic digestion technology can be used for treating organic wastes, such as solid slaughterhouse waste, to combine material recovery and energy production (Beal et al. 1999). Nutrient extraction recovers nutrients in a concentrated form (e.g., the inorganic precipitate struvite) from waste and can be desirable for providing a wider range of options for eventual reuse with reduced pathogen risk and improved ease of transportation (Mehta, et al. 2015). Aerobic-activated sludge systems can be used for wastes (e.g., for paper pulp sludge) to reduce the volume, chemical oxygen demand (COD), and biochemical oxygen demand (BOD) (Turner et al. 2022). Sometimes controlled incineration is used for high-risk wastes with the resultant ash being available for application to land (Ragasri, and Sabumon 2023).

**Table 2:** Selected industrial wastes with pretreatment options

Waste type	Pretreatment method/technology	Reference
Abattoir waste	Rendering	Mittal 2006
	Composting	
Wood ash	Hardening or granulation	Maresca et al. 2019
	Pelletising	Hannam et al. 2016
Dairy effluents	COD removal of fat	Karpati, et al. 1990
	Aerobic digestion	Wendorff 2001
		Van Horn etal. 1994
Tannery wastes	Fermented hydrolysates, treatments with mineral fertiliser, vermicomposting	Rigueto et al. 2020
Sugar beet pulp	Extract nano elements, bio-adsorb contaminants from wastewater	Rana et al. 2022
Paper pulp sludge	Aerobic activated sludge systems, aeration, and mixing to oxidise, or a combination of these or other methods to generate a secondary sludge	Turner et al. 2022; Veluchamy and Kalamdhad., 2017; Monte et al. 2009

There are several reviews in the literature that cover the assessment of waste pretreatment options for different types of waste (e.g., Mittal 2006; Rigueto et al. 2020). Thus, we refer readers to these reviews for a more in depth understanding of how waste pretreatments can be utilised to overcome some of the potential risks associated with applying wastes to land, and we do not directly discuss waste pretreatment further here.

### Waste effects on soil physiochemical properties

#### Soil texture

Soil texture has an important influence on the physiochemical behaviour and management of wastes and their constituents (Table 3). For example, between 65 and 95% of fly ash particles have a diameter of less than 0.01 mm, which generally resembles a silt loam soil (a preferred texture in agricultural situations) (Pathan et al. 2003a). If well managed, the incorporation of wastes, such as fly ash, into soil may alter the texture favourably, which in turn influences soil properties such as hydraulic conductivity (Basu et al. 2009; Chang et al. 1977). Impacts of waste applications on soil texture are likely to impact on other soil physicochemical characteristics including hydraulic conductivity, soil aeration, and nutrient and contaminant bioavailability (Dexter 2004), and thus, is discussed further in the relevant sections below.

**Table 3:** Waste application to land and effects of wastes on soil physiochemical properties

Soil property	Waste type	Outcome	Reference
Soil moisture/water movement and retention	Fly ash	Increase bulk density, increase porosity, increase hydraulic conductivity	Basu et al. 2009; Chang et al. 1977; Ghodrati et al. 1995a; Ghodrati et al. 1995b; Moreira et al. 2016; Pandey et al. 2009; Pathan et al. 2003a; Young et al. 2008; Zhai and Horn 2018
	Dairy effluents, meat processing effluents	Change/reduce hydraulic conductivity	Balks et al. 1997; Sparling et al. 2015
	Coal fly ash	Reduce hydraulic conductivity	Pathan et al. 2003b
Soil pH	Bauxite, cement, and lime kiln, fly ash, sugar beet lime and wood ash, dairy waste, steel slag and paper pulp	Liming agent to reduce acidity	Aitken et al. 1984; Allen et al. 2007; Liu et al. 2011; Cline et al. 2000; Jala and Goyal 2006; Mäkelä et al. 2012; Manoharan et al. 2010; Qing et al. 2015; Spark and Swift 2008; Tarkalson et al. 2005
Redox potential	Black coal fly ash, tannery waste	Changes in redox effecting solubility and mobility of contaminants	Chuan and Liu 1996; Gupta and Sinha 2006; Kumpiene et al. 2008; Miretzky and Cirelli 2010
Organic matter	Fly ash, tannery sludge, dairy effluents	Mostly increased organic matter, decreased in some cases	Cline et al. 2000; Gupta and Sinha 2006; Manoharan et al. 2010; Roy and Joy 2011; Singh et al 2011; Spark and Swift 2008; Sparling et al. 2001
Soil salinity and sodicity	Tannery waste, dairy wastes, pulp and paper mill effluent, fly ash	Increase soil salinity and/or sodicity	Alvarez-Bernal et al. 2006; Fisher and Scott 2008; Hawke and Summers 2003; Johnson and Ryder 1988; Kumar and Singh 2003; Liu et al. 2011; Manoharan et al. 2010; Matsi and Keramidas 1999; Punshon et al. 2002; Roy and Joy 2011; Singh et al. 2012; Wong et al. 1995

#### Soil moisture, water movement, and retention

Optimal soil composition is balanced between water movement and retention to ensure adequate aeration, provide sufficient water for plant and microbial growth, and drain excess water from the soil matrix. Changes to the physical characteristics of a soil due to waste applications can influence soil attributes related to water infiltration, water retention, aeration, and drainage. For example, the addition of fly ash to an undescribed “garden” soil at rates of 25%, 50%, and 100% (w/w) decreased the bulk density from 1.3 to 1.2, and 0.9 g cm^−3^, respectively. Inversely related to bulk density, soil porosity improved from 24 to 33%, 42%, and 60% respectively (Pandey et al. 2009). Increased porosity enables the retention of pockets of water and air and establishment of microhabitats for soil organisms and enhanced root penetration (Moreira et al. 2016; Young et al. 2008; Zhai and Horn 2018).

Clay soils characteristically retain more water than other soil types, which can lead to unfavourable soil conditions for agriculture due to waterlogging. Increased hydraulic conductivity can reduce waterlogging and its effects on the soil environment. Several studies have cited enhanced hydraulic conductivity after mixing fly ash (Chang et al. 1977; Ghodrati et al. 1995a; Ghodrati et al. 1995b; Pandey et al. 2009; Pathan et al. 2003a), dairy factory effluent (Sparling et al. 2015), and meat processing effluent in soils (Balks et al. 1997; Matheyarasu et al. 2016a, b). For example, soil permeability initially decreased by more than 50% when a silt loam soil was treated with meat processing effluent (Balks et al. 1997). Whilst this initial reduction was significant, it then gradually increased over 41 days, to the point where there was no significant difference in permeability from the original sample levels (Balks et al. 1997). In comparison, an experiment investigating water retention measured in situ in sandy soils amended with weathered, fine (< 20 µm) black coal fly ash identified a 25% reduction in hydraulic conductivity (Pathan et al. 2003b). Ultimately, the impact of waste amendments on porosity and subsequent aeration and hydraulic conductivity of soils is related to the characteristics of both the waste and the receiving soil.

#### Soil pH

Soil pH has an important impact on the solubility, binding potential, and speciation of elements and potential pollutants (Reichman 2002; Sauve et al. 2000). Soil pH subsequently affects the bioavailability of nutrients and contaminants to plants and soil organisms (Menzies et al. 2007; Reichman 2002). An optimum soil pH, typically favoured for most agricultural practices, is within the range of 6 to 7.5, with the specific optimum pH varying according to the crop type (DPI 2005; NLWRA 2001). Industrial wastes with an alkaline pH have the potential to be used as liming agents for moderating the pH of acidic soils (Jala and Goyal 2006; Spark and Swift 2008; Tarkalson et al. 2005). Provided that other potential environmental impacts are balanced, industrial wastes with prospective liming properties provide an opportunity for cheap lime substitutes, for example, bauxite, cement and lime kiln, fly ash, sugar beet lime, and wood ash (Allen et al. 2007).

The dynamic nature inherent in soil environments and the inconsistent composition of wastes mean their efficacy as liming agents can be highly variable. A comparison between several class F fly ashes and CaCO_3_ added to acid soils showed the ash was between 1/15 and 1/20 as effective as the CaCO_3_ in raising the pH of acidic sandy and loamy soils with a pH < 5.5 (Aitken et al. 1984; Manoharan et al. 2010). A similar study revealed class F fly ash applied at a rate of 0.8–3.2% (w/w) in a sandy loam soil raised the pH between 0.2 and 0.4 units, compared with a commercial liming agent applied at a rate of 0.2% (w/w), which raised the pH by 0.75 units (Cline et al. 2000). A greenhouse pot trial involving two soil types (Podzol and Ferrosol) showed that over 8 weeks, ten times the weight of fly ash was needed to raise the soil pH to the same level as lime (Spark and Swift 2008). Fly ash also typically has high concentrations of metals,^2^ and thus the need to use greater mass of fly ash to achieve the same pH as with the lime, meant an increase in soil metal concentrations in the fly ash treatment (Basu et al. 2009). Consequently, any derivative of coal sourced from class F material, and other wastes, should undergo thorough evaluation when considering applying it to agricultural lands as a liming agent. Other industrial wastes such as wood waste (Bang-Andreasen et al. 2017), dairy wastes (Liu et al. 2011), steel slag, and paper pulp (Mäkelä et al. 2012) when applied to land have shown liming effects in soil similar to commercial ground limestone (Mäkelä et al. 2012; Qing et al. 2015).

Changes in soil pH can affect the chemical equilibrium and subsequently, the mobility of substances in the soil. Iron and manganese oxides, which are particularly susceptible to fluctuations in pH levels, form stable compounds in aerobic soils, adsorbing, and thereby immobilising, many potentially toxic soil contaminants (Gadde and Laitinen 1974; Johnson et al. 2016; Tabassum et al. 2015). As a result of this immobilisation, repeated industrial waste applications may result in accumulation of contaminants within the soil profile (McKenzie 1980; Wuana and Okieimen 2011). For example, As mobility and bioavailability in the soil environment is highly dependent on soil pH and redox potential (Al-Abed et al 2007). A batch leaching experiment by Al-Abed et al.,(2007) showed that 98% of the As released from Fe-rich mineral processing waste was associated with Fe-oxyhydroxides and oxides (Al-Abed et al 2007). Under aerobic conditions, an increase of pH from 3 to 7 correlated with a decrease of As and Fe concentration in solution from 150 to 950 µgL^−1^, respectively, to approximately 30 µgL^−1^ each, due to the precipitation of Fe-oxyhydroxides (Al-Abed et al. 2007). The maximum solubilisation occurred at pH 11, with solubilised As at 3592 µgL^−1^ and Fe at 1683 µgL^−1^ (Al-Abed et al. 2007). Thus, the amendment of waste materials requires sound scientific knowledge of both the wastes and soils involved to strategically manage solubility, bioavailability, and mobility of potentially toxic compounds as pH changes.

#### Redox potential

The oxidation/reduction (redox) potential of a soil is an important factor in the solubility, mobility, and bioavailability of metals and other contaminants (Fiedler et al. 2007; Fijałkowski et al. 2012; Aigberua et al. 2018). Reducing conditions occur in low oxygen, anaerobic situations such as waterlogging, soil compaction, and the rapid decomposition of organic matter (Goldberg and Smith 1984; Hamon et al. 2004; Kaur et al. 2020; Patrick and Jugsujinda 1992; Reichman 2002; Rothe et al. 2016). The redox state affects the availability of metal contaminants that may be present in industrial waste directly and indirectly by affecting the solubility, speciation, and toxicity of metal species in the soil solution (Schwab and Lindsay 1983; Olaniran et al. 2013; Wuana and Okieimen 2011).

Changes in redox condition can have a direct effect on the valency and speciation of metals in soil (Pardue and Patrick 2018; Roberts et al. 2005). For example, Cr, a common element in steel, alloy, and tannery wastes (Miretzky and Cirelli 2010), is typically found in two oxidation states in soils: Cr(III) and Cr(VI) (Alvarez-Bernal et al. 2006; Bartlett and Kimble 1976). Under increased aeration and change in pH, Cr(III) is oxidised to Cr(VI) (Apte, et al. 2006). An analysis of Cr in tannery sludge identified Cr(III) as the predominant species, with Cr(III) and Cr(VI) concentrations of 5.02 mg/L and 0.25 mg/L, respectively in the leachate of the toxicity characteristic leaching procedure (TCLP) (Chuan and Liu 1996). Whilst Cr(III) tends to be the predominant species in soils, well-aerated soils often have Cr present as the more toxic Cr(VI) (Jardine et al. 2011; Landrot et al. 2012). As the speciation of elements can vary with redox conditions, the impact of redox conditions on the speciation of certain elements is an important consideration in assessing the risk of applying wastes to land. This is particularly so because of the potential for soil redox conditions to change with time (e.g., intermittent waterlogging) possibly changing the speciation, solubility, mobility, and bioavailability of elements from the waste.

Changes in redox conditions can also indirectly affect the solubility and mobility of bound contaminants. Soil colloids, such as clay minerals, hydrous oxides, and organic matter, immobilise contaminants via sorption. For example, Fe oxides concentrated on the particle surface of fly ash have the potential to sorb metal contaminants and reduce their bioavailability (Kumpiene et al. 2008). Manganese and Fe oxides have great capacity to sorb trace elements (Suda and Makino 2016). However, Fe and Mn oxides are reduced under anaerobic conditions resulting in solubilisation of the metals in the oxides (Olomu et al. 1973; Patrick and Jugsujinda 1992; Quantin et al. 2002). When metal oxides are solubilised, the trace elements previously sorbed to the metal oxide surface become dispersed into the soil solution, potentially resulting in metal toxicity or increased mobility (Basu et al. 2009). For example, soils treated with tannery waste have been found to contain Cd, Cr, Mn, and Zn bound to Fe and Mn oxide complexes (Gupta and Sinha 2006). Changes in redox conditions in the receiving soil could result in changes to the bioavailability of these contaminants. Thus, when wastes are added to soils, it cannot be assumed that redox conditions and the associated speciation, solubility, and toxicity of trace elements will remain unchanged over time.

#### Organic matter

Organic matter has a number of important roles in soil, including providing a source of nutrients, as an energy source for soil organisms, buffering soil pH changes, conserving soil structure, and regulating water holding and aeration characteristics (Simpson and Simpson 2012; Bot and Benites 2005). Furthermore, the bioavailability and leaching of metal ions, pesticides, and other contaminants can be reduced by the formation of stable complexes with organic matter (Bolan and Duraisamy 2003; Bonin and Simpson 2007; Brady and Weil 1996; Reichman 2002). Sometimes, incorporating waste products into the soil can improve the amount and retention of organic matter in soil (Corti et al. 2012). For example, dried tannery sludge mixed with dried, unspecified “garden” soil at five rates of 10 to 100% showed an increase in organic matter from the control by 1.0 to 10.1%, respectively (Gupta and Sinha 2006). In comparison, a study using fly ash at rates of 5 to 40% (w/w, equivalent to 50 to 400 t ha^−1^) found a decrease in the organic C of the sandy loam soils despite the addition of 10% farmyard manure in each treatment (Roy and Joy 2011). At a rate of 40% w/w fly ash, Roy and Joy (2011) recorded a maximum loss of 29% organic C compared to the control-untreated soil. Singh et al. (2011) observed similar trends of decreased organic matter in soils treated with fly ash. However, contrary to these findings, Punshon et al. (2002) successfully increased soil organic matter over 3 years from 3.1 to 5.2% after treating kaolinite clay soils with up to 1120 Mg ha^−1^ of fly ash. Sparling et al. (2001) found a moderate decline in the total C and N content of soil, irrigated with dairy factory effluent, which was accompanied by an increase in microbial biomass of more than double that of the untreated control. Thus, the reason for declines in total organic C after addition of waste amendments to soil may be due to increased microbial activity.

In the long-term, organic matter is not static, but is in a constant state of flux between formation and decomposition. Therefore, unless organic matter containing wastes are regularly added to soil, any impacts (positive or negative) are likely to be relatively short term in nature.

#### Soil salinity and sodicity

Some industrial wastes have the potential to increase soil concentrations of sodium and other soluble salts to toxic levels (Balks 1998). Unfortunately, little research on industrial wastes has directly investigated the impacts of salts in wastes on soils, and so, most of the results presented here are secondary outcomes of the research rather than the focus of the study. Nevertheless, the body of work suggests that some wastes applied to land pose a risk of increasing soil salinity and sodicity.

In general, soils are considered saline when the electrical conductivity (EC) of the soil solution exceeds 4 dS m^−1^ (Munns and Tester 2008; Sposito 2008). However, moderately sensitive plants can be adversely affected when the EC of the soil approaches 2 dS m^−1^ (Brady and Weil 1996). Alvarez-Bernal et al., (2006) investigated agricultural soils irrigated over 25 years with water polluted with tannery and other industrial wastes. A comparison of industrial wastewater-irrigated clay soils with well-water-irrigated clay soils on nearby farmland found industrial wastewater contributed to an increase in EC from 0.6 to 2.3 dS m^−1^ (Alvarez-Bernal et al. 2006). Other wastes found to heighten soil salinity and sodicity include pig slurry (Brechin and McDonald 1994), pulp and paper mill effluent (Johnson and Ryder 1988), dairy wastewater (Fisher and Scott 2008), and fly ash (Manoharan et al. 2010; Punshon et al. 2002; Roy and Joy 2011; Singh et al. 2012; Wong et al. 1995). For example, the application of fly ash into clay soils at a rate of 50 g kg^−1^ soil increased the EC from an initial 0.5 to 2.5 dS m^−1^ (Matsi and Keramidas 1999). An assessment of fly ashes sourced from several locations across Australia found a diverse electrical conductivity ranging from 0.14 to 19.1 dS m^−1^ (Yunusa et al. 2011). However, wastes with a high calcium content, such as alkaline fly ash, may assist in the amelioration of sodic soils (Yunusa et al. 2006). Following the application of alkaline fly ash at a rate of 7.5 kg per 100 kg soil, the exchangeable sodium potential (ESP) in a sodic, sandy loam soil was reduced from 14.8 to 10.4% (Kumar and Singh 2003). According to Hawke and Summers (2006), farm effluents contain high concentrations of K. Therefore, the application of farm effluents such as dairy effluents to land has the potential to increase the concentration of exchangeable cations in the upper soil profile (Hawke and Summers 2003) and increase soil salinity. These examples demonstrate how application of industrial wastes can impact positively and negatively on soil salinity and sodicity depending on the sodium and calcium concentrations in the wastes.

The reduction of soluble salts, as well as other elements, commonly found in wastes has been successfully demonstrated by weathering wastes, a process that reduces salt concentration by natural or artificially induced leaching (Allinson 2008; Finkelman et al. 2002; Ghodrati et al. 1995a; Pandey and Singh 2010; Punshon et al. 2002; Yunusa et al. 2011). The washing of red mud, a by-product in the production of alumina extraction from bauxite, with deionised water for 1 h resulted in a significant reduction of chloride, fluoride, sulphate, and vanadium from 4880 mg L, 32 mg L, 1140 mg L, and 555 µg L to 128 mg L, 16 mg L, 530 mg L, and 485 µg L, respectively (Brunori et al. 2005). However, there are major limitations to the weathering process. Firstly, the weathering and leaching of waste require substantial amounts of water (Yunusa et al. 2011). Furthermore, the leaching of waste constituents can cause groundwater contamination and is not specific to contaminants but can also leach essential nutrients, limiting the nutrient potential of wastes (Yunusa et al. 2011). Finally, even if wastes contain salts at low concentrations, a tendency for salts to accumulate in soil over time requires long-term management plans to accommodate recurring waste applications without compromising soil and plant productivity. Consequently, weathering wastes to reduce salt content may not be feasible as a practical method of treating wastes prior to soil application unless managed carefully, including the potential for off-site impacts.

## Contaminants

Many anthropogenic wastes are a complex mixture of inorganic and/or organic chemicals that, if applied to soil in excess, can produce toxicity impacts in plants, livestock, ecosystems, and humans. Some of the potential contaminants and waste sources are summarised in Table 4.

**Table 4:** Categorisation of the types of contaminants found in wastes

Contaminant type	Waste type	Typical contaminants present	References
Inorganic contaminants	Tannery waste	Cr, Cu, Pb, Sr, V, Zn	Gowd et al. 2010; Calheiros et al. 2007; Wang et al. 2004
	Farm effluents	Cu, Zn	Wang et al. 2004
	Dairy industry waste	As, B, Ca, Cd, Cr, Cu, Fe, K	Cameron and Di 2019
	Paper pulp	Zn, As, Ba, Cd, Pb	Shakil and Mostafa 2021
	Potable water treatment residuals	As, Mn, Cr	Sullivan et al. 2010; Turner et al. 2019; Verlicchi and Masotti 2000
Organic contaminants	Fly ash	PAH, PCB, PCDD, PCDF, and dimethyl and monomethyl sulphate	Shaheen et al. 2014
	Tannery waste	Chlorinated phenols, PCB, pesticides	Chowdhary et al. 2020
		Phenol, various dyes	Gomes et al. 2016; Piccin et al. 2013; Rigueto et al. 2020
Emerging contaminants	Dairy industry	Hormones, antibiotics, udder-cleaning antiseptics, and topical applications	Arvanitoyannis 2006; Fisher and Scott 2008; Schlüsener and Bester 2006
	Paper pulp	Hydroxy furanones with mono-, di-, and trichloromethyl groups	Franzén and Kronberg 1994
	Tannery wastes	PFAS mainly L-PFOS	Flores et al. 2013

*PAH* polycyclic aromatic hydrocarbons, *PCB* polychlorinated biphenyls, *PCDD* polychlorinated dibenzo-p-dioxin, *PCDF* polychlorinated dibenzofuran, *PFAS* per- and polyfluoroalkyl substances, *L-PFOS* linear-perfluorooctane sulfonate.

### Inorganic contaminants

There are many studies documenting increased metal loading and bioavailability in soils treated with industrial wastes (Alvarez-Bernal et al. 2006; Calheiros et al. 2007; Gowd et al. 2010; Lee et al. 2006; Roberts et al. 1994; Singh et al. 2010b; Mäkelä et al. 2012). Elevated metal concentrations, like Cr (2652 mg/kg), Cu (43 mg/kg), Pb (38.3 mg/kg), Sr (105.3 mg/kg), V (54 mg/kg), and Zn (160 mg/kg) (values are presented as means), were found in soil treated with tannery waste in India (Gowd et al. 2010). Calheiros et al. (2007) reported Cr accumulation by *Phragmites australis* and *Typha latifolia* after tannery waste application demonstrating the bioavailability and potential for food chain transfer of Cr in tannery wastes. Land application is a common option for farm effluent management. Farm effluents also contain metals, such as Cu and Zn but metals in farm effluents have received less attention than metals in other industrial wastes applied to land (Wang et al. 2004) due to the long history of applying farm effluents as soil amendments. These results highlight the potential for soils treated with industrial wastes to accumulate metals and the potential for these contaminated soils to result in metal enrichment of crops (Nan et al. 1999).

### Organic contaminants

There is little literature that has directly studied the impacts of organic contaminants in industrial wastes applied to land with some research on indirect application of organic contaminants to land e.g., via smelter fallout. The limited research on organic contaminants in industrial wastes applied to land demonstrates similar outcomes to those of metals in the soil environment. When introduced to soils, many organic pollutants undergo rapid degradation as a result of chemical and metabolic processes (Schnaak et al. 1997; Dabrowska et al. 2005). However, similarly to metals, contaminants categorised as persistent organic pollutants (POPs) resist environmental degradation and therefore can persist and accumulate in soils.

Coal fly ash contains various organic compounds including polycyclic aromatic hydrocarbons (PAHs), polychlorinated biphenyls (PCBs), polychlorinated dibenzo-p-dioxins (PCDDs), polychlorinated dibenzofurans (PCDFs), and dimethyl and monomethyl sulphate (Shaheen et al. 2014). Whilst the concentrations of PAHs and PCBs in fly ash are generally low, their effects on soil biota, uptake by plants and soil persistence still need to be assessed (Fig. 1). When fly ash is used in land application in excessive amounts or after multiple applications, it could pose significant risk to the wider environment (Shaheen et al. 2014). Tannery wastewater includes chlorinated phenols, PCBs, pesticides, and many more organic chemicals (Chowdhary et al. 2020). (Alam et al. 2010) Tannery wastewater application and the effects of associated POPs on soil have not yet been documented.

The risks of POPs in waste used as soil amendments are not thoroughly investigated in the literature. The inherent nature of POPs to persist in the soil needs to be considered if industrial wastes are to be sustainably utilised as soil amendments.

### Emerging contaminants

Previous research on utilising wastes as agricultural inputs has predominantly focused on the spread of pathogens, nutrients, and metal concentrations in plants, soils, and groundwater (Fig. 1) (Jjemba 2002a). New contaminants are emerging that have only recently been recognised as pollutants with potential impacts on the environment and human health. Emerging contaminants are defined as potentially hazardous inorganic or organic compounds that were previously not considered or known to be present in the environment (Brindha and Schneider 2019), and safe environmental thresholds are generally not covered by existing government policies or are still in the process of being refined. Emerging contaminants include hormones (e.g., estradiol and testosterone), chemicals from firefighting foams (e.g., per- and polyfluoroalkyl substances (PFAS), plastics (including microplastics and nanoplastics), pharmaceuticals (e.g., melengastrol acetate and trenbolone acetate), and industrial cleaning agents, which may contain potentially toxic sequestering agents and surfactants (Arvanitoyannis 2006; Bilal et al. 2021; Shi et al. 2021; Sweeney 2002). The bioavailability of emerging contaminants is largely governed by the physiochemical properties of the soil environment, the dosage applied, and the inherent properties of the chemical (Fisher and Scott 2008; Jjemba 2002a). Many emerging contaminants bind strongly to soils and organic matter, thus inhibiting their mobility and prolonging persistence in the soil environment (Hildebrand and Farenhorst 2006; Jjemba 2002a). However, some veterinary and industrial compounds in soils are more mobile and are therefore capable of contaminating ground or surface water (Stumpe and Marschner 2010) or becoming bioavailable to plants and soil biota (Jjemba 2002b). Once the contaminant is mobile, there remains uncertainty as to the long-term fate and effects of emerging contaminants on ecosystems. Whilst many agricultural and veterinary chemicals have beneficial applications that enhance productivity and control destructive pests and diseases, various environmental pollutants including hormones, pharmaceuticals, and surfactants can be present in sludge and wastewater used for irrigating soils (Orlando et al. 2004; Rowland et al. 1997). The dairy industry is a prominent user of veterinary chemicals, with dairy wastewater typically containing proteins, salt, fatty substances, lactose, and residue chemicals including hormones, antibiotics, udder-cleaning antiseptics, and topical applications (Arvanitoyannis 2006; Fisher and Scott 2008; Schlüsener and Bester 2006). Estimates of yearly estrogens and androgens excreted by farm animals in the USA and Europe show cattle to be the principal source of both hormones, with dairy cow slurry containing up to 500 times more oestrogen than bull slurry, followed by swine, sheep, and chickens (Allinson 2008). Concerns have been raised about the application of dairy farm wastewater as a fertiliser in agricultural landscapes after high concentrations of hormonally active agents were identified in Australia (Allinson 2008; Shore and Shemesh 2003). Animal manures have also been cited as a possible source of antibiotics and synthetic steroid oestrogens (Gadd et al. 2010; Kumar et al. 2005; Gudda et al. 2022; Li et al. 2021). The introduction of antibiotics into soil environments fertilised with pig manure (Schmitt et al. 2006) and poultry manure (Chaves-Ulate et al. 2021) increased the number of bacteria displaying genes resistant to that particular antibiotic. An experiment on six antibiotics commonly used in agriculture, and therefore likely to be present in agricultural wastes, investigated antibiotic persistence within a sandy loam soil over 120 days (Schlüsener and Bester 2006). A half-life of 5 to 27 days was calculated for five of the antibiotics (salinomycin, tylosin, tiamulin, erythromycin, and oleandomycin), but roxithromycin had only slightly decreased in the soil by the end of the 120-day duration of the experiment and therefore, no half-life could be determined (Schlüsener and Bester 2006). Tannery wastes have been found to be contaminated with PFAS mainly L-PFOS (linear-perfluorooctane sulfonate) (Flores et al. 2013) but, to the authors’ knowledge, the effects of tannery waste application on PFAS in soil are still not documented. In addition to a lack of knowledge of chemical fate and behaviour, there is little known about the dynamics with other chemical residues present in soils, such as pesticides (Fisher and Scott 2008; Hayes et al. 2006). Limitations remain as to the potential use of a number of industrial and agricultural wastes as soil amendments because of the presence of emerging contaminants. Further studies are needed to investigate emerging chemical fate, behaviour, and toxicity in soil environments before a conclusive response can be made about emerging chemicals of concern in waste materials applied to land.

## Offsite impacts

### Ground water contamination

Wastes added to land can adversely modify soil properties in ways that impede the proficiency of soil processes to immobilise and degrade soil contaminants before reaching groundwater. For example, batch experiments found leaching of Cr from chromite ore processing waste was dependent on soil pH and organic matter content (Weng et al. 1994). Once wastes are added to agricultural soils, the potential exists for contaminants and nutrients present in the wastes and soils to leach into groundwater and associated human drinking water sources such as wells (Kumar et al. 2007; Yadav et al. 2002). Metals do not undergo microbial or chemical degradation, therefore persisting and potentially accumulating in soils and the wider environment after repeated waste applications to soil (Bolan and Duraisamy 2003; Kim et al. 2009). Metals commonly accumulate in the soil surface due to the strong affinity of the soil matrix for these compounds (Cameron et al. 1997). Eventually, the concentration of metals may exceed the holding capacity of the soil and increase bioavailability, runoff to surface water and/or leaching into groundwater. In comparison, other potential contaminants may not be so tightly bound to soil, for example, nitrates and sodium chloride (Brady and Weil 1996), and are more likely to leach into groundwater or be transported in surface runoff. In a field-study by Yadav et al. (2002), traces of nitrate, Pb, and Mn were found in a well near farmlands irrigated with wastewater and sewage effluent, suggesting that the groundwater supplying the well was contaminated by the waste treatments nearby. Similar results were found in a study in Tamil Nadu, India with salts present in the tannery waste effluent found to leach into groundwater sources (Kumar et al 2007). Due to the high salt content in the tannery waste effluent, the crops no longer grow when irrigated with this water (Kankaria et al. 2011; Madejón et al. 2001). Also, high salt and P concentrations in dairy wastes have been reported to result in groundwater contamination when applied to land (Liu et al. 2011). Similarly, organic contaminants entering the soil typically undergo volatilisation, mineralisation, and/or leaching that may transfer contaminants into the groundwater (Semple et al. 2003).

High rainfall or a large application of high-water containing wastes can increase the downward movement of contaminants through the soil profile and potentially into groundwater. A column experiment with a loamy sand soil (pH 6.4 to 6.7) amended with two different fly ashes at a 30% (w/w) ratio showed Zn, Cu, and Ni initially leached rapidly from one particular fly ash type with reductions from 12, 16, and 4 mg L^−1^ respectively, to < 0.5 mg L^−1^ each after 25 cm of rainfall (Ghodrati et al., 1995b). The second fly ash type also had rapid Zn leaching after the first 30 cm of rainfall, from 5.6 to < 0.5 mg L^−1^, but leaching of Cu and Ni was insignificant. This initial high leaching rate of some metals suggests that early stages of waste application will have the greatest leaching potential before the more soluble forms of metals have leached and remaining metals have sorbed onto the soil matrix. Therefore, the initial high leaching potential of metals from wastes could potentially be managed by staggering waste applications. Furthermore, evaluation or assessment of waste leaching potential needs to account for rates of leaching over time, in addition to the total contaminants leached. In total, the proportion of metals leached after 150 cm of rainfall from ash-amended soils averaged 14% of the metals added to the soil (Ghodrati et al. 1995b). Although 14% may not seem excessive, when considered in the context of the agricultural industry over a large land area, this may equate to a concerning total mass of metals entering groundwater.

Few studies have evaluated the leaching potential and impact of organic contaminants introduced directly to soils through industrial waste application. Organic contaminants entering the soil may undergo mineralisation and/or leaching processes that can result in contaminants moving from the soil and into groundwater (Semple et al. 2003) and is an area of research requiring more attention.

### Surface runoff and impacts on waterways

Inadequate mixing of wastes into the soil matrix and/or poor surface plant cover can cause problems such as erosion and contamination of surrounding soils and water (Basu et al. 2009). Thus, mixing wastes thoroughly into soils is important for the homogenisation of wastes and their constituents and reducing erosion and surface run off. Most of the solid manure and liquid waste from concentrated animal farming operations is applied to croplands as the final method of waste disposal. Due to excessive application or inadequate mixing of these wastes with soil, the nutrients can end up in storm water runoff and water bodies causing high concentrations of N and P in waterways (Heckrath et al. 1995; Harter et al. 2002). For example, wastewater from dairy industries often contains high concentrations of P and N that can cause algal blooms in waterways (Arvanitoyannis 2006; Sampat et al. 2018). Researchers are attempting to address this problem by reducing the protein levels in animal feeds to limit N excretion (Belloir et al. 2017; Vieira et al. 2016). A study investigating irrigation with poultry waste and its effects on P loads to Lake Tenkiller, USA, found that applying poultry waste to land had increased the P load to the lake from 311 t year^−1^ to more than 528 t year^−1^ with increased potential for eutrophication (Jeon et al. 2015). Furthermore, hormones and other veterinary therapeutic agents in dairy effluent and wastewater may have negative impacts on aquatic life (Miracle and Ankley 2005; Pal et al. 2010; Smital et al. 2004). Fish and other aquatic vertebrates are susceptible to toxicity from oestrogens, with concentrations as low as 1 ng L^−1^ in waterways causing negative impacts (Stumpe and Marschner 2010).

## Effects on soil biota

### Soil microbial populations and functioning

Depending on waste- and soil-related variables, some microbial communities may favour waste-induced environmental changes whilst other communities may decline (Cameron et al. 1997). Even moderate waste-induced changes to the soil environment, including pH, salinity, and accumulation of contaminants, can have significant impacts on soil microbes (Barajas Aceves 2005; Broos et al. 2007; Huang et al. 2009; Roy and Joy 2011; Yunusa et al. 2011). For example, the number of protozoa in soils treated with chloroquine and quinacrine dihydrochloride, therapeutic agents commonly found in animal wastes, were increased compared to the control soils from 78 soil to 180 protozoa g^−1^ soil respectively (Jjemba, 2002b). In contrast, the lowest concentration of metronidazole at 0.5 mg per g^−1^ soil reduced the protozoa in the rhizosphere tenfold (Jjemba 2002b). A study by Roy and Joy (2011) showed fly ash at rates of 50 and 100 t ha^−1^ benefited soil microbial populations, probably due to the nutrients added to the soil. However, fly ash applications exceeding 100 t ha^−1^ correlated with a decrease in colony numbers of soil bacteria, fungi, and actinomycetes (Roy and Joy 2011). Hayat et al. (2002) investigated microbial organisms in soils containing elevated metals accumulated over 12 years of wastewater irrigation. A standard spread plate test on metal tolerance of various functional groups (aerobic heterotrophs, asymbiotic nitrogen fixers, actinomycetes, and fungi) in metal concentrations of 200 µg ml^−1^ for Ni, Cd, Pb, Co, Cu, Cr, Zn, and Hg showed moderate to high tolerance in all groups possibly suggesting adaptation to high metals from long-term exposure. However, microbial survival rates were shown to be reduced significantly at 400 µg ml^−1^ of metals (Hayat et al. 2002). This correlation between increasing rates of waste and decreased microbial biomass has been found in a number of other studies (Barajas Aceves 2005; Fliessbach et al. 1994; Giller et al. 2009; Huang et al. 2009), and is suggestive of the negative impact on soil microbes and the occurrence of toxicity thresholds of contaminants introduced into soil via wastes.

The modification of soil microbial populations and activity can significantly impact ecological functions important for sustainable agriculture such as nutrient cycling and organic matter decomposition. Microbial sensitivity to metal stress and the subsequent reduction of soil functions has been well documented (Giller and McGrath 1989; McGrath et al. 1988, 1995). For example, Roy and Joy (2011) reported a dose-dependent decline in amylase activity (as a measure of microbial polysaccharide decomposition) in soils treated with fly ash at ≥ 50 t ha^−1^, which appeared to recover within 60 days in soils treated at 50 and 100 t ha^−1^ fly ash. For higher applications of fly ash (200 and 400 t ha^−1^ fly ash), the reduction in amylase activity persisted beyond the 60 days of the experiment (Roy and Joy 2011). Similar trends were found for other microbial enzyme activities (cellulase, invertase, dehydrogenase, and arylsulphatase) under laboratory and field conditions (Roy and Joy 2011). Alvarez-Bernal et al., (2006) found contrasting findings with wood ash application to soil. Bacterial numbers significantly increased up to a wood ash dose of 22 t ha^−1^ followed by significant decrease at 167 t ha^−1^ wood ash application to soil. Wood ash application changed the soil bacterial composition, with copiotrophic bacteria responding positively and oligotrophic bacteria negatively. Alvarez-Bernal et al., (2006) also found a trend of decreasing bacterial richness and diversity as the wood ash application rate increased. In comparison, soil microbial biomass was increased in other studies with applications of tannery waste (Nakatani et al. 2011) and dairy waste (Liu et al. 2011).

Waste constituents amended into soils are likely to change the soil microbial communities, but the evidence suggests that genetically diverse microbial communities, at least initially, are more able to adapt and maintain broad functions, although with a different species and genetic composition in the soil (Alvarez-Bernal et al. 2006; Giller et al. 2009; Johnson and Ryder 1988).

The disparity in microbial responses to contaminant loading rates and site-specific factors are so diverse that it is difficult to generalise outcomes based on specific test results. Nevertheless, some microbial functions such as nitrification are necessary for plant growth, and thus agriculture, so further research into these outcomes is important to ensure sustainable waste application on agricultural lands.

### Plant production

#### Benefits to plant production

By providing nutrients or altering soil properties, wastes can potentially enhance soil fertility and plant production. Experiments have demonstrated notable results with fly ash amendments at rates ranging from 5to 20% fly ash to soil dry weight ratio, resulting in benefits to seed yield, increased growth performance, produce and quality of crops including mung bean (*Vigna radiata*) (Singh and Agrawal 2010), pigeon pea (*Cajanus cajan*) (Pandey, peanut (*Arachis hypogaea*) (Mittra et al. 2005), rice (Dwivedi et al. 2007; Lee et al. 2006; Mittra et al. 2005; Singh et al. 2012), maize (*Zea mays*) (Spark and Swift 2008), centipedegrass (*Eremochloa ophiroides*) (Adriano et al. 2002), and ryegrass (*Lolium perenne*) (Matsi and Keramidas 1999). Studies have attributed the positive growth outcomes to increased nutrient loading in soils treated with wastes (Giachetti and Sebastiani 2006). A study in Australia found an increase of 2.5 to 4.5 times the extractable-P in soils amended with fly ash compared with untreated soil (Pathan et al. 2003b). The study attributed the increase of extractable-P to the direct addition of fly ash, noting an earlier experiment that found fly ash contained 92.5 µg g^−1^ extractable-P (Pathan et al. 2003a, 2003b). Estimates suggest a moderate application of fly ash at 10 t ha^−1^ could potentially provide up to 110 kg P ha^−1^, an amount with the potential to support crop P requirements for 3 to 5 years if the P is all in bioavailable forms and does not leach out of the soil profile (Yunusa et al. 2011). Glass house demonstrations show fly ash added at rates of 5 and 25 t ha^−1^ enhanced growth and yield of canola (*Brassica napus*) by approximately 18% compared with an untreated control soil (Yunusa et al. 2008). A study by Luo et al. (2004) investigating irrigation with meat-processing wastewater at an annual loading rate of 600 kg ha^−1^ of N over 2 years, resulted in an increase in plant growth in two silt loam soils due to increased nutrient loadings. Comparable results on growth and flower yield of *Chrysanthemum* plants were demonstrated using potted planting media amended with tannery waste (Singh et al. 2011). This study demonstrated that tannery waste at lower concentration promoted vegetative growth of *Chrysanthemum* cuttings whilst being a growth inhibitor at higher concentration most likely as a result of metal accumulation in plants (Calheiros et al. (2007).

#### Adverse effects on plant production

Although controlled waste applications may develop favourable soil conditions and provide essential nutrients to plants, excessive or recurring applications may introduce new constituents to the soil that eventually exceed threshold concentrations for plant health (Besen et al. 2021).

Maintaining metal concentrations within tolerable concentrations can promote vigorous plant growth in waste-treated soils. However, plants express a range of stress responses when elevated concentrations of metals exceed toxicity thresholds, including altered metabolism and growth reduction (Angulo-Bejarano et al. 2021; Jamla et al. 2021; Nagajyoti et al. 2010). Canadian poplar (*Populus euramericana),* grown in lysimeters that had been treated with 192 t ha^−1^ of tannery waste, had high metal concentrations in tissues, in particular Cr, which appeared to be the primary factor reducing plant growth in comparison with lower dose treatments (Giachetti and Sebastiani 2006). After 4 months, *P. euramericana* appeared to be overcoming the impacts of the waste despite initial symptoms of growth retardation and some foliage chlorosis (Giachetti and Sebastiani 2006). Whilst the mechanisms for plant recovery were not specified, overcoming this large application demonstrates the potential for utilising large quantities of waste if other issues are adequately addressed.

It is likely that the adverse effects on plant productivity of some wastes are caused by the absorption of added contaminants by plants. Whilst it is worth noting the potential for plants to overcome soil toxicity, applying wastes with the intention of soil amelioration can have detrimental impacts on plant growth if poorly managed. Therefore, potential adverse effects must be considered in addition to known benefits when considering waste applications.

#### Plant bioaccumulation of contaminants

Crops tolerant of elevated concentrations of contaminants pose different problems compared to more sensitive species because of their potential to accumulate metals (Nagajyoti et al. 2010; Sayyad et al. 2010) and other contaminants (Dobslaw et al. 2021; Lesmeister et al. 2021; Zhang et al. 2021), which may ultimately bioaccumulate and enter the food-chain (Puschenreiter et al. 2005). Tolerance of chemicals in plants varies based on plant species; contaminant type and concentration; and the soil physiochemical environment. An increase in metal loading in the edible portion of plants grown in waste-treated soils has been demonstrated for a range of crops and metals (Kim et al. 2009; Kumar et al. 2007; Pandey et al. 2009; Punshon et al. 2002; Singh et al. 2010a; Yunusa et al. 2008). Higher rates of fly ash resulted in increased concentrations of B, Cu, and Mo in the leaves of canola plants at flowering and of Mo in the grain (Yunusa et al. 2008). High concentrations of B and Se were also noted in several species of pasture and turf grass grown on fly ash-amended soil (Matsi and Keramidas 1999; Pathan et al. 2003b; Punshon et al. 2002). Although less studied, the bioaccumulation of organic and emerging contaminants has also been identified in plants grown in waste-amended soils (Jjemba 2002b; Kipopoulou et al. 1999; Migliore et al. 1996, 1995). Thus, the potential exists for land application of wastes to result in bioaccumulation of chemicals from the waste in plants and needs to account for potential bioaccumulation and associated health risks.

## Waste application effects on human and animal health

The potential for contaminants from wastes applied to land to enter the food chain is largely dependent on the initial concentration of contaminants in wastes, rate of waste application, and soil properties affecting bioavailability, especially pH, organic matter, and clay content (Bolan and Duraisamy 2003). There have been few studies on food-chain transfer of contaminants from soils treated with waste to farm animals raised for human consumption. Cadmium impurities in fertilisers applied to land in New Zealand have been shown to result in bioaccumulation of cadmium in the livers and kidneys of grazing animals (Roberts et al. 1994). Whilst fertilisers are not industrial waste, the presence of cadmium bioaccumulation in these animals suggests that elevated concentrations of metals from industrial wastes applied to land may have similar effects. Studies in India detected higher metal concentrations in milk samples from dairy pastures irrigated with wastewater in comparison to samples from clean water-irrigated pastures (Singh et al. 2010a). Although contaminant concentrations were relatively low, milk samples from cows eating wastewater-irrigated pastures contained three times higher cadmium and nickel, five times higher copper, lead, and zinc, and seven times higher chromium concentrations than milk produced under control conditions (Singh et al. 2010a, b).

In addition, there have been minimal studies documenting accumulation of contaminants from wastes applied to land on non-agricultural animals, such as invertebrates, birds, mammals, or reptiles. A number of general studies (i.e., not specific to land application of wastes) documenting the bioaccumulation of POPs in plants, animals, and animal produce (Polder et al. 2010; El-Shahawi et al. 2010; Jones and De Voogt 1999), which have been attributed to the chemical persistence and lipophilic nature of POPs (Kipopoulou et al. 1999). The potential health risks posed by emerging contaminants entering ecosystems and the food chain are a contentious area, and there is little direct evidence from research on land application of wastes published. Several studies have demonstrated endocrine-disrupting effects of various chemicals Alsen et al. 2021; Allinson 2008; Annamalai and Namasivayam 2015; Colborn et al. 1993; Ismail et al. 2017; Kiess et al. 2021; Muñiz et al. 2017; Sharma et al. 2020; Yang et al. 2015). However, available research on emerging contaminants has primarily focused on sediment and aquatic life, with reports of hormonal changes, such as variations in production of gender-specific proteins, occurring in fish in waterways contaminated by cattle effluent (Allinson 2008).

The application of waste to agricultural lands poses the risk of contaminants passing through the food chain, and thus, affecting human food products (Xiaobin et al. 2021). For consumer safety and public support of industrial waste as a soil amendment, it is important that we increase our knowledge of the potential for contaminants in wastes applied to land to transfer to livestock and associated products (meat, eggs, milk) via the food-chain.

Bioaccumulation of contaminants within the food chain and contaminated residues on the outside of food (particularly vegetables and fruits) are two of the major routes of human exposure to contaminants from wastes applied to land (Chary et al. 2008; Hashmi et al. 2021). Singh et al. (2010a, b) assessed agricultural produce grown on soils irrigated with wastewater from surrounding industries, including dye, plastic, recycling, and metal surface treatment facilities. Of the 16 plant species tested for Cd, cabbage, rice, and wheat had the highest risks with hazard indices of 10.2, 9.2, and 5.9, respectively. Lead was highest in wheat (*Triticum aestivum*) (4.37), rice (*Oryza sativa)* (6.8), and cauliflower (*Brassica oleracea* var. botrytis) (7.5), whilst Ni was highest in wheat (2.4), cabbage (*Brassica oleracea* var. *capitata*) (1.8), and amaranthus (*Amaranthus* sp.) (1.6) (Singh et al. 2010a, b). In comparison, Cu, Cr, and Zn indices in these crops were found to be well below 1.0. The result from this study illustrates the potential for risks from consuming food produced on land amended with industrial wastes, and there is an urgent need for further research in this area.

Human health risks posed by wastes applied to land may also include direct occupational exposure during or after the application process. This is particularly so if application to land requires additional transport and handling compared to direct disposal in local ponds or landfill. A United States Environmental Protection Agency (USEPA) assessment of the risks in handling fly ash found that due to the high extractability of metals in fly ash, extra care is required in handling the ash residue to prevent ingestion and absorption of ash particles across the human gastrointestinal tract (Manskinen et al. 2012). Inhalation of fly ash may also possibly contribute to various diseases like silicosis, fibrosis of lungs, bronchitis, pneumonitis, and may contain carcinogenic agents (Kravchenko and Ruhl 2021; Basu et al. 2009, Whiteside and Herndon 2018). Although no literature was found on the risks of direct industrial exposure of applying other industrial wastes to land, it is likely that some wastes may also pose similar handling and inhalation risks.

## Conclusion

There is significant potential for adding wastes to land as soil amendments. There are also potential negative environmental and human health impacts associated with applying wastes to land. Therefore, to reduce pollution and other negative impacts of applying waste to land, the preference is to reduce the production of industrial waste at the source and practise a more circular economy approach to industrial production. However, where that is not possible, the application of industrial waste to land has potential if it occurs within a risk-based and sustainable manner. Safe waste application to land is a combination of effective waste pretreatments and optimising the interaction between the physicochemical properties of the waste and the receiving soil. In this review, we concentrated on the interaction between waste characteristics and soil parameters and the potential benefits and risks associated with applying wastes to land.

Several soil parameters influence the bioavailability and mobility of waste contaminants once added to soil, including soil texture, pH, organic matter, and redox conditions. An appreciation of the importance of soil properties and the diversity of soils is important for the sustainable management of waste applications within the agricultural sector. The current literature demonstrates the potential for the application of industrial waste into agricultural soils and highlights the prospects of linking sustainable waste management with farming practices. However, there is a lack of comprehensive and conclusive findings in direct relation to industrial waste being applied to agricultural soils. Long-term and mass balance assessments are needed to investigate the long-term physical, chemical, and biological outcomes of industrial wastes in agricultural landscapes. Other areas in need of further research include risk profiling of soil types to determine which have the highest and lowest risks associated with waste application; assessment of potential on- and off-site consequences of sustained use of waste amendments; cost–benefit analyses; treatment of wastes prior to use as soil amendments; and risk assessments of the fate and behaviour of chemicals in wastes that have been applied to land. Ultimately, to understand the impact of wastes in agricultural soils, further studies are required that look at multiple applications and long-term impacts. Only recently has the research literature commenced critically evaluating the potential of beneficial waste applications in the agricultural sector, and we hope that our review encourages more research in this area.

## Data Availability

Not applicable.

## Footnotes

1 Fly ash is composed of fine solid particles made of ashes, dust, and soot produced by burning coal and other materials.

2 For simplicity, throughout this paper the term metal will be used to denote both metals (e.g., Cu, Zn, Ni, Pb) and metalloids (e.g., As, Sb).
